# Gender disparity in publication records: a qualitative study of women researchers in computing and engineering

**DOI:** 10.1186/s41073-021-00117-3

**Published:** 2021-12-01

**Authors:** Mohammad Hosseini, Shiva Sharifzad

**Affiliations:** 1 Feinberg School of Medicine, Northwestern University, Chicago, USA; 2 MAS,Graduate Institute of International and Development Studies, Geneva, Switzerland

**Keywords:** Gender disparity, Equity, STEM, Authorship, Qualitative research

## Abstract

**Background:**

The current paper follows up on the results of an exploratory quantitative analysis that compared the publication and citation records of men and women researchers affiliated with the Faculty of Computing and Engineering at Dublin City University (DCU) in Ireland. Quantitative analysis of publications between 2013 and 2018 showed that women researchers had fewer publications, received fewer citations per person, and participated less often in international collaborations. Given the significance of publications for pursuing an academic career, we used qualitative methods to understand these differences and explore factors that, according to women researchers, have contributed to this disparity.

**Methods:**

Sixteen women researchers from DCU’s Faculty of Computing and Engineering were interviewed using a semi-structured questionnaire. Once interviews were transcribed and anonymised, they were coded by both authors in two rounds using an inductive approach.

**Results:**

Interviewed women believed that their opportunities for research engagement and research funding, collaborations, publications and promotions are negatively impacted by gender roles, implicit gender biases, their own high professional standards, family responsibilities, nationality and negative perceptions of their expertise and accomplishments.

**Conclusions:**

Our study has found that women in DCU’s Faculty of Computing and Engineering face challenges that, according to those interviewed, negatively affect their engagement in various research activities, and, therefore, have contributed to their lower publication record. We suggest that while affirmative programmes aiming to correct disparities are necessary, they are more likely to  improve organisational culture if they are implemented in parallel with bottom-up initiatives that engage all parties, including men researchers and non-academic partners, to inform and sensitise them about the significance of gender equity.

**Supplementary Information:**

The online version contains supplementary material available at 10.1186/s41073-021-00117-3.

## Background

Despite the increasing presence of women in research activities, their average publication records (including the number of published items and received citations) are still lower than those for men [1–3]. Although disparities vary depending on academic seniority and research disciplines [4], they impede women researchers’ career development [5], contribute to ethical issues by hampering gender equality [6] and distort institutional excellence in academia [7]. More recently, the COVID-19 pandemic and the resulting public health restrictions have also exacerbated some of these disparities [8].

Previous studies have provided a wide range of explanations for the disparity between women researchers’ and men researchers’ publication records. For instance, some studies acknowledge the challenges related to motherhood and caring responsibilities, including re-entry after a career break due to pregnancy, childcare, and other care responsibilities [9]. Others note that women are more engaged with teaching than research [10] and are often hired on part-time and temporary contracts [11]. Furthermore, women are less likely to request or be offered a (better) position in the authorship byline [4] or cite their own published work [12]. Although revealing, these factors do not explain the disparity in all contexts and academic environments, and might affect researchers’ publication records in other academic environments differently.

Our initial literature search of this topic showed that there is limited qualitative information about prevailing gender issues in Irish universities (details available in the supplementary document). A rare example pertains to the qualitative research conducted by Linehan and colleagues on organisational practices that reproduce gender inequality in University College Cork [13]. This study suggested that given the slow pace of change between 1985 and 2010 in terms of women’s representation in senior academic positions, gender issues should be better prioritised and (junior) women researchers should become more informed and speak up about gender discrimination in their environment.

Given the aforementioned considerations, our first goal was to fill the knowledge gap, and to contextualise gender disparity in publication records at DCU’s Faculty of Computing and Engineering. Our second goal was to investigate factors that, according to women researchers, have contributed to their lower publication records.

### Initiatives and policies aimed to improve gender equality in Ireland and at DCU

**Fig. 1:**
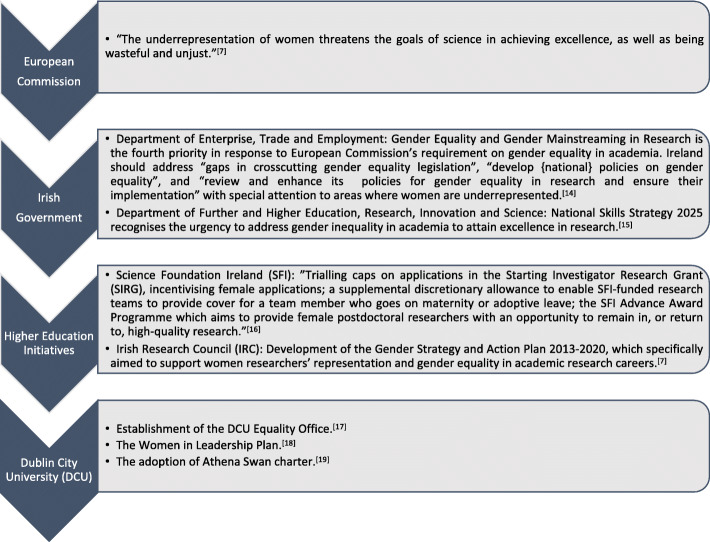
Institutional stances on gender policies in Ireland and at DCU

In the early 2000s, the European Commission stated that “the underrepresentation of women threatens the goals of science in achieving excellence, as well as being wasteful and unjust” [7]. Subsequently, the Irish government prioritised gender equality in academia, resulting in the adoption of strategies by higher education authorities such as Science Foundation Ireland (SFI) and the Irish Research Council (IRC) to remove barriers to women’s participation in academia (Fig. 1) [14–16].

**Fig. 2:**
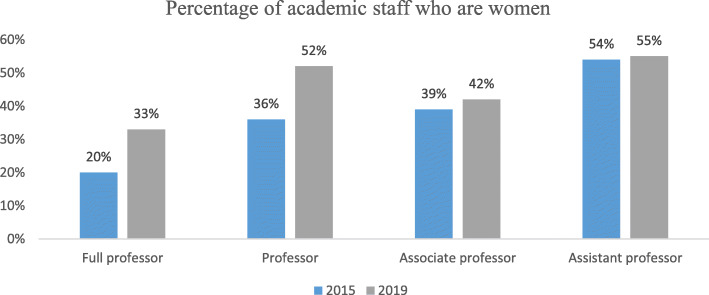
According to The Talent, Discovery, and Transformation: Strategic Plan 2017–2022, between 2015 and 2019, the percentage of women academic staff across DCU grew in every grade (equivalent academic titles up to 2016 were professor, associate professor, assistant professor and lecturer). The report presents this data in a table and the current graph was produced for this paper

Consistent with the general policy of the Irish government and initiatives promoted by SFI and IRC, DCU has adopted policies to promote gender equality. DCU’s Equality Office monitors “processes and procedures within the University to ensure equality of opportunity” between all members of the community [17]. DCU announced its Women in Leadership Plan in 2015 “with a view to addressing the issue of gender inequality at higher grades of appointment in the University” [18] and introduced a wide range of initiatives to realise this vision. Consequently, as demonstrated in Fig. 2, between 2015 and 2019, the percentage of academic staff who identify as women across DCU increased at every grade [18].

The Women in Leadership Plan also supports research on gender issues with a focus on enhancing women’s representation in academia (e.g., return to work after leave and gender equality in the recruitment and selection processes) [18]. Following its efforts to enhance gender equality since 2017, DCU is one of the thirteen higher education institutions in Ireland (out of a total of twenty-six) to have achieved the Athena SWAN Bronze award [19].

**Fig. 3:**
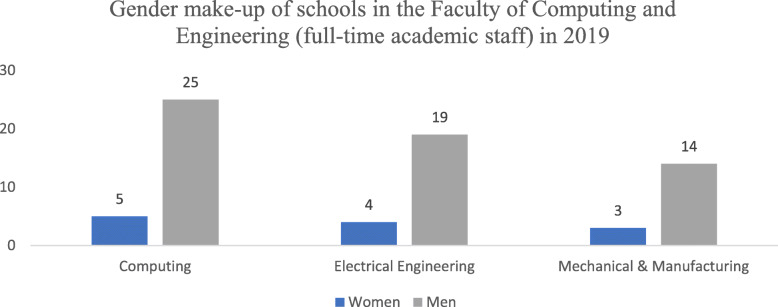
The gender make-up of the academic staff, as reported by the DCU Research Office (excluding PhD candidates and postdoctoral researchers) in the Faculty of Computing and Engineering in 2019

Despite such initiatives, gender disparity still exists in some areas at DCU. Indeed, despite the increased representation of women between 2015 and 2019 and also the appointment of a woman to the post of Executive Dean for the Faculty of Computing and Engineering, the gender make-up of academic staff in 2019 (excluding PhD candidates and postdoctoral researchers) was still dominated by men (Fig. 3). Additionally, our exploratory quantitative study showed that, across the Faculty of Computing and Engineering at DCU, women researchers have fewer publications (per person in a five-year period), collaborate less often in international projects, and receive fewer citations than men [20].

## Materials and methods

This study started with an exploratory quantitative analysis (details available in the supplementary document) to compare the publication records of men and women researchers affiliated with DCU’s Faculty of Computing and Engineering over a five-year period (2013–2018). The quantitative analysis echoed what other studies had suggested about an overall lower average in the publication record of women researchers [1, 2], and showed that similar trends are noticeable at this faculty. It should be noted that these disparities are partly explained by a higher concentration of men researchers in senior positions, and the presence of hyper-productive and well-cited men in the sample (the top two authors who identify as men published 236 and 157 items, respectively, in a five-year period, and received 797 and 1415 citations for these items, respectively).

Given the importance of publication and citation records for career development and resource access [21, 22], exploring these metrics could shed light on sub-optimal representation of women in academia. The rationale for choosing the Faculty of Computing and Engineering was that overall, in Europe, and also at DCU, these areas continue to be heavily dominated by men. According to the latest SHE Figures report, only 29% of doctoral graduates in engineering, manufacturing and construction are women [23]. At the time interviews were being conducted, only 17% of staff in DCU’s Faculty of Computing and Engineering were women [20], suggesting that gender issues could be more pronounced in these disciplines and, as a result, require more urgent attention.

In reporting the study, we followed Consolidated Criteria for Reporting Qualitative Research (COREQ), and the checklist is presented in the supplementary document [24].

### Participant invitation and recruitment

The research protocol and the first draft of the semi-structured questionnaire were developed based on the results of the quantitative analysis and several published reports, including Elsevier’s *Gender in the Global Research Landscape* [25] and the European Commissions’ *She Figures* [23]. Later, two experts (one based at DCU and one external) and the Dean of the Faculty of Computing and Engineering at DCU provided suggestions that improved descriptive and Likert type questions. Subsequently, the questionnaire was tested in a pilot and finalised after further adjustments. The DCU Research Ethics Committee granted approval in April 2019 (REC Reference: DCUREC/2019/081). Participants were recruited via email (with the help of the heads of the Schools of Computing, Mechanical and Manufacturing Engineering and Electronic Engineering) and via direct contact (using the available list of employees on the Schools’ websites). Since bulk invitations sent by the heads of the Schools only resulted in two positive responses after two weeks, direct email invitations were sent to thirty-three women researchers using the publicly available list of researchers affiliated with each School. A reminder email was sent after two weeks to non-respondents.

### Interviews

We received sixteen positive responses. Sociodemographic information of the interviewees is presented in Table 1.

**Table 1:** Sociodemographic information of 16 interviewed women at DCU’s Faculty of Computing and Engineering

Personal Information	Number (***N*** = 16)
Married	6
Has children	4
Irish citizen	5
European citizen (non-Irish)	2
Full-time contract (not necessarily permanent)	16
PhD candidate	7
Postdoctoral researcher	3
Assistant professor	4
Associate professor	2

All interviews were conducted in person by M.H. in DCU but in a building other than where the participants worked in order to protect their anonymity. Interviews took place between May and September 2019 and were audiotaped. All interviewees were briefed on the research aims and objectives, received the printed information sheet and informed consent form to sign (see the supplementary file), and were given a chance to ask questions about research objectives and methods before the interview started. Interviews varied in length from 22:29 to 53:45 min and lasted an average of 36:13 min. At the start of each interview, interviewees were briefed about the results of the exploratory quantitative analysis. They were reminded about their own publication and citation record as detailed by Google Scholar and Scopus. At the end of each interview, the interviewer read fifteen statements and asked interviewees to react using five Likert type responses (i.e., strongly agree, agree to some extent, neither agree nor disagree, disagree to some extent, strongly disagree). Responses to these questions are presented in the supplementary document.

### Analysing interviews

The recordings were replayed and transcribed in January 2020 by a non-author contributor who was recruited as an intern through the DCU INTRA Office. During the transcription process, any information that could identify participants was obscured or removed. Subsequently, both authors coded the interviews using an inductive approach, as suggested by Thomas [26]. We started the analysis by coding three randomly selected interviews. We then compared results and developed a codebook. After discussing the first set of codes, we agreed on an initial list of fifteen category codes and used them to analyse the rest of the interviews. Upon analysing all interviews, and after further discussions, most of the codes were refined and revised. This led to the development of three new codes and the removal of five codes and overlap reduction (some codes were merged). This brought the total number of codes to nine (both versions of the codebook are available in the supplementary document). Using these codes, we analysed all interviews for a second time. After the first draft of the results was completed, we sought feedback from an external reviewer (experienced in qualitative research methods) and improved our analysis. Furthermore, when revising the manuscript based on the feedback provided by peer-reviewers, we refined the title of three codes, but this did not affect our coding.

In drafting the results section, we used responses to the Likert type questions that were directly related to the identified codes to complement and add more nuances to our analysis. Furthermore, we replaced terms indicative of language habits with recommended terminology for discussions about gender (i.e., men/women researchers instead of male/female researchers) [27]. In the discussion section, we employed a conceptual framework (i.e., intersectionality) to explore the differences between the identified issues and provide actionable recommendations. In preparing the manuscript for publication, we chose to delete the quote labels that represented the unique codes for each interview in order to protect the anonymity of our interviewees.

## Results

We found that the publication records of women researchers at DCU’s Faculty of Computing and Engineering were impacted by various factors. These factors could be subsumed under two headings:

1. *Factors that hinder women’s engagement in research publications,* including gender roles, implicit gender biases, negative perceptions of their expertise and accomplishments (by others), women’s (own) high professional standards, family responsibilities and nationality.
2. *Factors that support women’s engagement in research publications,* including networking and research communication, collaboration with other institutes, and funding acquisition.

### 1. Factors that hinder women’s engagement in research publication

At the start of each interview, interviewees were asked whether they were satisfied with the number of papers they had published. Fourteen said that they were not happy and that they wished they had published more. The two exceptions were both PhD candidates – one who noted that she had too many publications and would have preferred to have had fewer, higher-quality publications, and another who was satisfied with the number of her publications. Subsequently, interviewees were asked to provide their perspective about the lower average number of publications, focusing on the extent to which they perceived gender to be a determining factor. Although none of the interviewees believed that DCU is an unpleasant place to work for women, and none believed that they are supported unequally in publishing their work (confirmed by the Likert type questions they answered, available in the supplementary document), in describing their lived experiences, interviewees mentioned some insights linked to their gender. We categorized issues brought up by interviewees under six themes: gender roles, implicit gender biases, negative perceptions of their expertise and accomplishments (by others), their (own) high professional standards, and the intersection of interviewees’ gender and their familial status and nationality.

#### Gender roles

In discussing various aspects of their work, sometimes interviewees framed disparities in terms of roles or tasks which are more often expected of women. They recounted their experiences of being perceived as having certain characteristics because of their gender, leading to the conclusion that since they are better at conducting a specific task, they are expected to do it. Although one interviewee noted that administrative staff take notes in her group, seven interviewees mentioned that administrative tasks (e.g., minute-/note-taking, delivering organised financial reports of international trips) are among those tasks that women are expected to engage in more often than men. According to one interviewee, “we spend a lot of time with that admin work instead of spending our time on research”. Another interviewee stated that she was asked to input published papers of the whole group to the system (“so yeah, I was just putting it in for everybody basically, I’ve done that the first two years [of my appointment]”). Men, on the other hand, tend to “refuse to do those pieces of admin work because they are too busy working on something else”. Furthermore, the organisation of training and monthly get-togethers sometimes frustrated women as described by an interviewee: “I never … I mean I never saw an instance where a man has been asked to arrange it next time”. These interviewees highlighted that women seem to be stereotyped as detail-oriented, well-organised and willing to help others.

The impression of three interviewees was that women are expected to work/deal with students more often than men. While one interviewee was not sure about the reason for being disproportionately approached by students (“they [students] just come [to the lab] and ask me loads of questions”), another interviewee suggested “they [heads of schools] do like to have women in roles in contact with students, because it is nicer for students to see women in the staff as well. And sometimes those student contacts can be very time consuming”. Another interviewee noted that contact with students could be distractive (“when you deal with students, there’s always admin issues”). Given the burden of administrative and organisational tasks, student support and teaching responsibilities, interviewees noted that it is difficult to focus on research and work on publications: “this [publishing] is important, but has low urgency, so loads of other things that are urgent tend to take over”.

In addition, one interviewee framed the burden of gender balance policies as an extra demand that negatively effects women’s publication record (because in departments with a disproportionately high number of men, on average, women are more often asked to join committees and boards): “If you’re trying to have the gender balance in terms of your committee makeups and your PhD examiners and all that, maybe there are higher demands on women because they’re trying to make up for this gender balance all the time”.

#### Implicit gender biases

In discussions about their publication records, participants described implicit gender biases that women face in professional environments, which indirectly affect their publications. Eight interviewees described these biases in terms of workload. Two participants felt that sometimes women are expected to work more, but were unable to explain why (“there are fewer demands from them because they are men”). Five other interviewees described the situation slightly differently, noting that they work late or at weekends because *they feel the pressure* to perform better than men, implying that the expectation is not necessarily imposed on them. Since “it is in people’s nature to think that men are really better”, women feel that they need to work harder or be “much more engaged to help or to suggest something or make more contribution[s]”. One interviewee noted that “women feel they need to prove a lot more and work a lot harder”. Another noted, women researchers “should be very ambitious and show that they can do everything like travelling or going for workshops anywhere in the world”. Two interviewees mentioned that they work overtime, even if it is not expected from them (“we are trying to be aware of gender and mental health as well, but I still feel guilty when I don’t work”). Another interviewee associated the higher workload with her skills (“they give me more work. I hope because I’m clever (laughter). I have more skills than the male researcher in my lab”).

Sometimes implicit biases were described in terms of how women are treated in specific situations. One interviewee felt that men are often assumed to be reliable professionals capable of doing excellent work, whereas if a “good logic” or “elegant solution” is presented by a woman, since it is not expected, it is considered unusual and “impressive”. Another interviewee noted that this perception (of lower technical competence) means that their mistakes “carry much bigger weight” than mistakes made by men, because a woman researcher who makes a mistake is considered as proof of the stereotype that she is incapable, whereas men researchers would be treated more charitably in similar situations. When it comes to being considered for more senior positions, “people may just assume that you might not want to get to senior positions. They might assume that you might want to have a lower stress role”. The perception that women who have family duties are less willing to work was believed to have a real impact on women’s involvement in collaborations and subsequent publications (“they think women are not involved with the work but with personal things, so they will not be willing to work more or they would not be willing to work overtime, but no, that’s not the case”). One interviewee believed these “unconscious biases” may be the reason why “we’re not getting enough women to these high levels, to these professorships”.

#### Negative perceptions of women’s expertise and accomplishments

Relevant to the previous theme, although more specific (i.e., cases wherein women’s expertise and professional accomplishments are negatively perceived), seven interviewees felt that because of their gender, their expertise is not always taken into account and their voice is not heard in (technical) discussions, thereby negatively affecting their engagement in projects and publications. When specifically asked about their DCU supervisors and mentors, none of the interviewees recalled being undermined or unfavourably perceived by them, but four participants mentioned having seen this attitude from other colleagues and partners from the industry. They noted that although opportunities for involvement in collaborative projects between academia and industry might be equal for men and women, “the industrial partner, they have lower expectations of women”. Having their expertise undermined was also mentioned in discussions about women’s citation records. One interviewee believed it is in people’s “nature” to assume that men do a better job compared to women, so they might overlook the research and publications where women are listed as first or last authors, thereby lowering the average citation record of women.

Two participants reported having had their achievements discredited by those who associated women’s success with gender equity policies. The first interviewee described a situation in which she received an award in her field, and instead of being praised, her success was brushed off by people saying “they wanted a woman anyway”. The second interviewee had similar experiences when she was invited to prestigious events (“I’ve been asked to speak numerous times and people were like ‘oh, they needed a woman’. Well, yeah, but I also do pretty good work”).

When asked about the Senior Academic Leadership Initiative (SALI) aimed to award forty-five senior positions to address women’s under-representation in academia [28], fourteen participants asserted that this is an important and urgent idea to compensate for women’s underrepresentation, yet most of them had reservations about it. One participant called it “a necessary evil”, while another said, “if it was totally equal [between men and women researchers] we wouldn’t need that help”. More importantly, only five participants felt entirely comfortable about accepting such a position. The rest anticipated that these promotions would be negatively perceived, and thus reinforce the stereotype about women’s alleged lack of competence, thereby negatively affecting women’s confidence and engagement in projects or publications. One interviewee said, “It is interesting that they want to have more women, this part is interesting, but if you pay attention it means that I cannot compete with men for other positions”. Another interviewee noted, “I can appreciate the motivation behind it. I don’t like the fact it’s necessary. People that I’ve spoken to have said that they think it probably is necessary, that you have to get that pinch point to make it normal, to make people see that it’s not strange to have women as professors and senior academics. But do I like the idea of it? No! Do I want to be a recipient of one of them? No!”.

#### High professional standards

As a general rule, when talking about their academic career, interviewees were quite confident that they have worked hard and have good knowledge of their field. However, some of the same individuals also said that their work is not “good enough”, that “they are not the most qualified”, and that sometimes their success was all down to “luck” or perhaps gender equity policies.

Although four interviewees acknowledged that securing more senior positions often provides better access to resources and results in more publications, they believed that they are less likely to apply for new positions than men researchers, as they do not feel confident about their work and are too demanding of themselves when submitting an application. According to one interviewee, “I was encouraged to apply for it [new position] this year, but I didn’t feel comfortable because I didn’t think I had enough publications”. Another interviewee described this as a general difference between men and women:

If there was a job going and a man was looking at it and he could do 20% of things at the requirements, he’d go: Oh, yeah, I can do 20%, I’ll be able to do the rest of the things, so I put myself forward. Whereas a woman might look at it and she’ll be like: There’s 20% that I can’t do from that list, I don’t know if I’d be suitable so I don’t put myself forward.^1^

According to another interviewee, “I think we take maybe more time to get a solution, but once you get the solution it’s going to work”. This suggests that some women may take more time to publish a piece that is satisfactory for them and, therefore, they may publish less often. A similar perspective was offered by another interviewee about conference presentations: “Men tend to submit more papers, because they believe it’s good, so maybe they get to travel more than women”. Two interviewees believed that the pressure in academia to publish more impacts upon research quality, suggesting that the focus on quantity over quality results in not appreciating some women researchers who have high standards and pay special attention to the quality of their publications.

Women’s high standards were also mentioned by interviewees who had worked with both men and women mentors, noting that some women supervisors might put more emphasis on quality or set strict learning objectives for mentees, thereby prolonging the required time to publish a paper for themselves and mentees (“She has more expectations from me than my man supervisor. I guess she is stricter and more particular and ambitious”). Another interviewee noted, “My woman supervisor is more like ‘okay, you can do that maybe’ or she just gives you a few ideas, but without [immediately] telling you what to do. And my man supervisor is more like ‘yeah, you have to do that’”. This suggests that her man supervisor is more easy-going with his mentees. One interviewee described higher standards in mentorship in terms of engagement and communication. Compared to him, she is “much more engaged to help or to suggest something”. Furthermore, another interviewee noted the differences in mentors’ approaches to authorship attribution, saying, “she asked me to be very careful in who I put as a co-author. Like, if someone did no work then they wouldn’t be a co-author. Right now it’s a little bit of a matter of friendship or just because there is a PI somewhere else, you may have to put his name [in the authorship byline]”. In an effort to explain women supervisors’ higher standards, one interviewee noted,

I guess that any of the women PIs that I’ve come across, who would be at the kind of senior level, tend to be exceptional. They tend to have excelled significantly. Some men PIs don’t seem to have as much drive. I feel like some of the men counterparts maybe haven’t had as much adversity to get to that point. So, therefore they may not have the same level of drive or determination.

#### Family responsibilities

Ten participants acknowledged the difficulties of balancing family and professional responsibilities and advocated for women researchers with (young) children. The intersection of family responsibilities and gender were mostly described in terms of motherhood. One researcher made a direct link between having young children and her publications (“I think that’s the main reason that I have fewer publications these years”). She added that one of her friends who was based in another Irish university ended up leaving academia and went to the industry because “when she did the interview [for a lectureship position], the examiner asked her a publication question and why she didn’t have publications in the recent years. Well, she had two kids during these years and she’s super busy with family and research, so she didn’t get the lecture position.” Two interviewees highlighted the challenges of travelling for mothers (“A woman who has a child couldn’t be away, maybe two or three days would be too long because of her child”; “woman researchers with kids wouldn’t have much support to travel”).

The impact of family on work and publications was not the same for all women. One interviewee noted that she shoulders the social demand placed on her by what she called “traditional norms”, which hold women responsible for taking care of the house and the family. She advocated for women in academia who come from traditional backgrounds wherein “husbands or fathers, they don’t help, their duties are different, and they have more freedom”. Another interviewee noted that although her husband is equally involved in childcare responsibilities, she feels more emotionally engaged:

Women still may be the primary nurturer of a child, maybe just their thoughts or they are more likely to be affected by it. Maybe that is more of an effect on women than it is on men, even if there is more kind of gender balance in childcare.

That said, two interviewees highlighted the flexibility of working hours in academia as an advantage:

I could work around [interviewee’s child] being off school and everything; so I think from that point of view, academia is a really good place to work. You wouldn’t necessarily get the same opportunity in industry.

#### Nationality

The intersection of respondents’ nationality and gender were mainly mentioned by non-Irish and non-EU interviewees (*N* = 11). Participants thought that being non-Irish and non-European has a negative impact on their involvement in projects and conference attendance, both of which influence their publications. Nine interviewees who were non-European citizens pointed out regular difficulties such as dealing with immigration services, learning about specific rules that apply to their situation, and figuring out support mechanisms for maternity leave.

Non-Irish interviewees felt that their chances of getting into a new position or getting involved in a new project are lower than their Irish counterparts, even if their experience and competencies were similar or slightly higher. One interviewee spoke of the experience of her non-Irish friends who had trouble securing an academic position at another Irish university:

My friends, one is [a European nationality] and another is [a non-European nationality]. They are very good at their area. They have a lot of publications and teaching experience; they are quite qualified for a lecturer position. Once they applied, they were told to do a teaching presentation. When Irish candidates finished presenting, everyone was just clapping, and said, “oh, so good”, but when non-Irish candidates finished presenting, nobody was clapping and nobody said anything. Yeah, but for their work experience, they actually had more publications than that Irish candidate and they had been in the post-doc position much longer than him as well. But finally the Irish person got the position.

The same concern was also raised by a non-Irish participant about the positions offered through SALI (“they will get all of them from Ireland”). Citing logistical difficulties and higher costs, one Irish interviewee confirmed some of these suspicions, noting that when:

The project needs to start soon, we need to get someone on board and the visas and issues in getting someone from a non-EU country here, logistics are harder. So, probably, if we have equal candidates of equal merit, we’d go with the European or the Irish … the other thing is the fees aspect for EU versus non-EU.

Non-Irish researchers noted that sometimes they struggle with being heard and appreciated in academic environments. One non-EU researcher was frustrated because her receipt of a prestigious grant was not promoted on the DCU website. She felt that smaller achievements of Irish researchers are under the spotlight more regularly. As highlighted by two interviewee, sometimes past achievements are disregarded because of nationality (“They [colleagues] say like ‘oh, you’re from [country], you guys have nothing there so like … is the education even good? Can I trust your grades that came with you?’”; “They feel like [the interviewee’s nationality] are all kind of poor or not educated or something”). Another interviewee said, “I have noticed in different groups for example, that if you’re not Irish, you can’t talk with them”, suggesting that sometimes non-native, non-Irish persons are ignored in conversations.

### 2. Factors that support women’s engagement in research publication

We identify three factors that, according to the interviewees, may positively impact chances for publication: (1) networking and research communication; (2) collaboration with other institutions; and (3) funding acquisition.

#### Networking and research communication

The importance of maintaining good relationships with other academics, not only in DCU but also outside of it, was discussed by nine interviewees. Personal and international connections were believed to affect the communication of published work and improve chances for future collaboration and publication. Three participants said that it is the responsibility of the researcher to seek information about collaboration possibilities and that they need to enhance the visibility of their work to become well-known.

Responding to the Likert type questions, twelve interviewees agreed or strongly agreed that social/educational/training activities at DCU are equally welcoming to both men and women. One interviewee, who had worked in industry, described DCU as a more inclusive environment for women (“when I worked in industry, I didn’t play golf, so immediately I was side-lined … here, there isn’t anything like that”).

Three participants noted that communication of work through social media and public talks positively impacts future collaborations and the number of citations. This is an area where men researchers seem to be more fortunate:

He [colleague] told me that he thinks … if he gives one talk, people tend to pay attention a bit more and then he gets invited to more stuff … I don’t know, maybe a man, a white man speaking about stuff, it’s more reliable than a woman talking about it.

Another interviewee suggested that men researchers are more successful in the social media engagement (“[A man colleague] does lots of things that are really clever, really smart, he is really active on Twitter, so his paper is tweeted about”). Two interviewees who praised men colleagues’ activities on social media did not seem comfortable in promoting their work in a similar way.

#### Collaborations with other institutions

Responding to the Likert type questions, twelve interviewees agreed or strongly agreed that they have the same opportunity as their colleagues to be involved in international collaborations. Whilst acknowledging that “collaborations with other researchers” and institutions, especially “interdisciplinary projects”, are among the most effective ways of publishing more often and receiving more citations, three participants reported having a hard time in certain projects, particularly those that involve international travel or working with industry partners. Two interviewees mentioned that in collaborative projects, it is mostly men who get to present their work in international conferences (“it’s more pressure for me, you know? I don’t have time to travel. I’m working for the same conference, but the guy went to the conference. I just have less time than him to prepare my abstract”; “Friend of mine (...) she has the name on the paper as well but her supervisor asked men researchers to present in a conference”). One of these interviewees believed that preferences for the involvement of men might be due to the idea that men “can handle themselves more easily” or that they are more flexible about the place to stay and the means of travel, thereby reducing the expenses for the project. Another interviewee mentioned that, in relation to collaboration with industrial partners, “sometimes for certain projects or company visits which are deemed a bit rough, the preference is to send men researchers”. Moreover, women researchers may be subject to a different communication style (“the industrial partner ( …) they’re kind of afraid of talking directly to a women researcher. And they may talk to the PI [Principal Investigator] about it and then ask the PI to talk to women”).

Other interviewees did not believe in systemic/institutionalised differences in research travel and collaboration between men and women. One interviewee noted that at some stage she was overwhelmed by the amount of travelling that she had to do for various international collaborations, and it is her own choice not to travel more. Another participant considered herself “fortunate” in terms of travelling for international collaborations, as she did not have any family duties or visa issues that non-EU residents have to deal with.

#### Funding acquisition

Six interviewees discussed the importance of funding for research projects and its impact on recipients’ power in developing and coordinating projects. They noted that funding not only enables “work”, “experiments” and recruitment of PhD candidates and postdoctoral researchers, but also empowers them in collaborative projects in terms of making decisions about subsequent publications (e.g., choosing the target journal, co-authors, authorship order). Four interviewees noted that as a “rule”, funding recipients also secure the better (last) position in the byline. Three participants mentioned the importance of funding for research travel, international partnerships and the visibility of their work (“I had funding enough to go and put myself in conferences, and you know, I did webinars and everything … so you get noticed”).

One interviewee mentioned the difficulties of receiving funding, especially for early-career researchers:

To get grants you need to have a good track record. You need to have the good ideas and you need to show that you are able to follow through, and get money and get things packed tight … I compete with those who are more likely to deliver because they’ve done it before; so it can be quite challenging just to kind of, get off the grind and get up and running.

This suggests that successful funding applications would increase the chances of getting funds again. Another participant suggested that although gender is not a decisive criterion for funders, the eligibility criteria in securing funds create a cyclical disparity (“You can’t just look at publications in isolation, you have to link [it] to the funding that people have [which allows them to publish]”). She noted that women are less likely to be awarded grants because the eligibility criteria for grants do not take into consideration their obstacles for publication. This issue was further elaborated by another interviewee: “if you’re compared with the person who doesn’t have any career break, it will be difficult”.

## Discussion and recommendations

Our study has shown that women researchers in the Faculty of Computing and Engineering at DCU face obstacles that may negatively affect the number of their publications. Whilst these obstacles might impact each individual differently, addressing different obstacles might also require different approaches. To explore the differences between the identified obstacles and enable this research to provide actionable recommendations, we will employ a conceptual framework commonly known as intersectionality to provide a holistic view of disparities and highlight the gaps in gender equality initiatives adopted by DCU or policymakers in Ireland. According to this framework, different examples of inequality can be subsumed under institutional, structural and interpersonal levels.^2^

*Institutional level inequality* concerns policies and rules adopted by government bodies or universities that are explicitly discriminatory against one group. In the context of this paper, institutional inequalities would entail those imposed on women researchers by higher education authorities and institutions. For example, until the early 1970s, Irish women had to resign from their work in the public sector when they got married [7]. This discriminatory rule was repealed in 1973 and inequalities of this type were not mentioned by any of the interviewees.

*Structural level inequalities* entail norms and rules which are prima facie gender-neutral but impact women differently than men. These policies and practices take no notice of women’s long-lasting underrepresentation in academia or specific obstacles related to their gender (e.g., motherhood), and, therefore, maintain or exacerbate inequalities by perpetuating a state of affairs that is less accommodating for women. The reported lack of gender sensitivity in funding criteria and hiring committees are illustrative examples, which show how gender-neutral criteria with no discriminatory intent can have different implications for women. Both funding organisations and hiring committees assess candidates based on academic excellence and competence, with publications as one indicator (meaning that there is no institutional inequality that excludes or limits women researchers). However, these gender-neutral approaches do not consider gender roles and biases or obstacles women face in attaining excellence and publishing their work, especially when they have career breaks or young children and happen to work in certain disciplines that continue to be dominated by men. In relation to funding acquisition, if women researchers do not have a competitive publication record, then they are likely to get stuck in a vicious circle of not having a competitive publication record because they do not have access to necessary funds, and not getting the necessary funding because they do not have enough publications. This should explain why existing research has reported that some women perceive gender as an influential factor in attainment of funding [29]. In relation to attaining more senior academic positions, women report experiencing difficulties in climbing the academic ladder because of having fewer publications, and they have fewer publications because they cannot climb the academic ladder as quickly as men.

One way of addressing these structural inequalities would be to revise success metrics and research assessment criteria in funding and hiring applications. Various strategies have been adopted in different European countries to counteract *the motherhood/parenthood penalty*, including eighteen-months expansion of the funding eligibility window for women per each new-born, flexible work time and mobility grants [30].

*Interpersonal (or individual) level inequalities* are about the interactions between women researchers and their colleagues. In the current context, gender roles, implicit gender biases, and negative perceptions of women’s expertise and accomplishments fall under this level of inequality. Although examples of interpersonal level inequalities could be considered as isolated and sporadic instances, as this research shows, they seem to have a lasting effect and negatively impact women’s confidence, professional standards and choices when applying for new positions or submitting their work for publication. As interviewees highlighted, even some attempts and initiatives aimed to promote gender equality could backfire because of existing interpersonal level inequalities. For example, while discussing SALI, eleven interviewees had second thoughts about accepting such positions, fearing that this might reinforce the belief that they are only successful in securing a position *because* of their gender, rather than being the most qualified for the position by virtue of their competencies. In other words, while SALI is aimed to address an acute structural-level inequality (i.e., the underrepresentation of women in senior academic positions), it was unpopular among some women researchers in our sample because of gender stereotypes that exist at an interpersonal level. We argue that these adverse reactions could result from an inadequate engagement with inequality at an interpersonal level and indicate an insufficient awareness about the significance of gender equality among men researchers and industrial/non-academic partners.^3^

Our point is that when initiatives to improve gender equality are implemented without adequate foreshadowing, which creates and promotes a culture that admires equality at an interpersonal level, well-intended initiatives could backfire (e.g., by reinforcing the stereotype that women are incapable engineers, hence, they need extra help). As mentioned by some interviewees, even an invitation for a talk could be perceived negatively (e.g., by saying, “oh, they needed a woman”) and further discredit the merits of women who accept such invitations. Other experiences mentioned in the interviews also elucidate the necessity of expanding the gender equality initiatives to interpersonal levels, especially in terms of delegating tasks. For instance, the perception that since women are supportive, detail-oriented and well-organised; they are better suited for tasks such as helping students, note-taking and organising training seems to associate individual capacities with expected roles and affect women both individually and as a group.

Furthermore, the stereotype that women researchers are less competent than men still persists in academia [31]. Although this view is not ingrained in any policy that directly discriminates against women, interviews showed that it has a tangible impact on how women position themselves in academia. The prevalence of such stereotypes, which are more noticeable in STEM fields [32], seem to have made some women (as the target group of stereotypes) to internalise them. Accordingly, they either identify themselves with the stereotypically incompetent researcher and hesitate to put themselves forward for conferences/positions,^4^ or they set very high standards for their work, spending much more time than men on their papers/applications to prove their competence and refute the stereotype. Either way, these attitudes show that because inequalities are not addressed at an interpersonal level, women are under pressure and feel that the onus is on them to make up for inequalities. In other words, the burden of responsibility is still on women as they seem to feel that they are *the problem* whereas, in fact, the prevalent culture of academic (and most notably STEM) environments seems to be the issue.^5^

We argue that the continued existence of these perceptions and stereotypes shows that while top-down approaches and policies are necessary, they also require complementary bottom-up initiatives that are designed to engage and create awareness among men researchers and industrial/non-academic partners. Among other solutions, existing research has recommended engagement with all employees and partners in discussions about gender equality by offering inclusion and diversity training [34]. That said, since various forms of these interventions (e.g., voluntary vs mandatory trainings) have received criticism in the literature [35], they should be considered carefully and their appropriateness assessed in relation to specific, concrete working environments. Given these complexities, efforts such as this research, which aimed to identify specific gender issues in one faculty, could inform tailored bottom-up initiatives and increase the likelihood of improving the organisational culture.

### Limitations and recommendations for future research

The small sample size limits the possibility of drawing general conclusions from this study. Furthermore, our quantitative analysis was exploratory and only gauged the average publication record of individual researchers between 2013 and 2018 without applying any normalisation method (e.g., academic age) or looking into other discrepancies (e.g., impact factor of journals where articles are published, self-citations, etc.). The conducted interviews only reveal the views of sixteen respondents and should not be regarded as representative. We did not interview men researchers as we were focused on reflecting the views of the underrepresented group (i.e., women). This study took a gender binary approach and has not enquired into or reflected LGBTQI+ concerns. Furthermore, all our interviews were conducted before the COVID-19 pandemic and do not reflect the challenges which resulted from public health restrictions and the socio-cultural effects of the pandemic in general. Some of the discussed themes (e.g., family and nationality) might negatively affect men researchers too. However, we argue that in the explored context, the intersection of family, nationality and other identified issues (e.g., gender roles, implicit biases and negative perceptions of women’s expertise and accomplishments) is likely to affect women to a greater extent. At the time of conducting the interviews, SALI was promoted as a women-only initiative, but recent changes to the eligibility criteria indicate that "in exceptional circumstances" men can also apply to these positions [28]. The impact of this change on how women researchers perceive the initiative is not reflected in our research.

Future research could explore experiences of women in other faculties at DCU or other universities in Ireland. Such investigations could focus on the experiences of women as a group, or experiences of specific cohorts based on, for example, career stage, familial state, sexual orientation and nationality. The intersection of these cohorts (e.g., a senior researcher who is a non-European single mother) creates unique experiences and challenges that deserve focused exploration. We recommend that specific attention be paid to the challenges faced by these cohorts (e.g., in hiring and tenure processes) and explore their lived experiences to determine whether academic environments are equally accommodating to all researchers. Using homogenous samples in terms of position/seniority could facilitate exploring gender inequality in different stages of an academic career. In addition, while comparative studies between different faculties of the same university could shed light on disciplinary nuances, comparison between men and women’s lived experiences could provide a clearer picture of the role of gender in academic environments.

## Conclusion

Exploring publicly available reports provided by higher education authorities in Ireland demonstrates that there have been numerous initiatives, on national and institutional levels, promoting women’s participation and representation in academia. However, analysing the lived experience of women researchers suggests that some areas still need improvement. In particular, our analysis highlighted issues related to gender roles, implicit gender biases, negative perceptions of women’s expertise and accomplishments, women’s high professional standards (resulted from the pressure to perform) and their family responsibilities and nationality as factors that might negatively impact women. Furthermore, gender-neutral criteria of funding organisations or hiring committees that do not take into account women’s specific conditions (e.g., career break, young children) are seen as factors that although possessing no discriminatory intent, negatively affect women.

These findings stress the need to expand gender equality initiatives, especially at an interpersonal level to address cultural and deep-rooted inequalities that affect women in academia. We suggest that gender equality initiatives should not be limited to affirmative actions (e.g., providing more positions for women researchers) and could be accompanied by, for example, tailored inclusion and diversity training informed by research, and the revision of success metrics and qualifications criteria in hiring processes and funding applications to account for the specific conditions of women researchers.

## Supplementary Information

**Additional file 1.**

## Data Availability

As part of the data management practice requested by the DCU Research Ethics Committee, the audio recordings and transcripts are retained until March 2021.

## Abbreviations

DCU: Dublin City University
HEA: Higher Education Authority
IRC: Irish Research Council
PI: Principal Investigator
SALI: Senior Academic Leadership Initiative
SFI: Science Foundation Ireland

## Footnotes

1 This view and some other opinions reflected in this section could be interpreted as implicit biases towards men. Although it may be true that some men would put themselves forward for a higher position regardless of their merits or fit for the function, or some men would not spend much time on their papers or be careless about the quality of their work, not all men adopt these behaviours and therefore, these attitudes should not be generalised.

2 These unique modes of inequality are inspired by discussions on intersectionality. In some texts, institutional and structural level inequalities might be used interchangeably, or structural inequalities might be subsumed under institutional ones. In this paper, we consider these as two separate levels. For more information on intersectionality see, e.g., Crenshaw, K. (2017). *On intersectionality: Essential writings*. The New Press, or Collins, P. H., & Bilge, S. (2016). *Intersectionality*. Polity Press.

3 While it seems hard to deny that the women hired via this initiative are being hired partly *because* of their gender, the aggressive affirmative action seems morally justified as corrective to gender inequality and biases of the past, some of which still persist. One can reasonably argue that for a long time, it was men researchers who were positively discriminated in hiring processes and had better chances of collaboration *because* of their gender. Nevertheless, since they were not stereotyped as incompetent, they did not feel uneasy about their privileges.

4 This issue is discussed in more detail by studies that suggest the imposter syndrome is more prevalent and severe amongst women researchers in academia [33].

5 This issue is also echoed by Ireland’s Higher Education Authority (HEA), which encourages the academic institutions to amend the organisational culture rather than following the ‘fix the women’ approach. According to the Report of the Expert Group which conducted the HEA National Review of Gender Equality in Irish Higher Education Institutions: ‘the Expert Group realised that the ‘fix the women’ approach (where women would change to fit the existing culture) usually adopted to try and solve gender inequality has not resulted in substantial change. Instead it is clear that organisation and culture must change in order that talented women, and others who do not automatically benefit from the status quo, are fully recognised and rewarded. Changing the organisational culture in this way will not hinder quality. A fair and transparent organisation will encourage women to have confidence that they will be recognised and assessed based on their true merit and excellence without unconscious bias, and in turn encourage more women to stay in the career pipeline.’ [7]
