# Supplementary material for: Gender disparity in publication records: a qualitative study of women researchers in computing and engineering
Source: Res Integr Peer Rev. 2021 Dec 1;6:15. doi: 10.1186/s41073-021-00117-3 (PMC8632200; doi:10.1186/s41073-021-00117-3)
Supplement: Supplementary file 1 — Additional file 1. [file 41073_2021_117_MOESM1_ESM.docx]

# Supplementary material

## **Scoping Search**

In January 2019, a scoping search was conducted using Google Scholar to find studies that interviewed faculty members of an Irish university to explore gender biases and disparities in academia. For this purpose, the following search string was used: “qualitative” AND “Ireland” AND “university” AND “gender bias” OR “gender disparity”. This search string yielded 6900 results. Reviewing the title and abstract of the first 200 items (the first 100 items sorted based on relevance and the first 100 items sorted based on date), it was concluded that the qualitative research conducted by Linehan and colleagues on organisational practices that reproduce gender inequality in University College Cork was the only relevant example.

## **Exploratory quantitative study**

The quantitative analysis aimed at comparing the publication of men and women researchers affiliated with DCU’s Faculty of Computing and Engineering (Figures 1, 2, 3). For this analysis, a list of research staff and their gender was requested from the DCU Research Office in May 2018. Subsequently, researchers’ publications between 2013 and 2018, the number of times these items were cited (as per August 2018) and co-authors’ affiliations were extracted from the Scopus database. Although publication disparity could be partly explained by a higher concentration of men researchers in senior positions, and the presence of hyper-productive men in the sample (the top two men authors published 236 and 157 items in a five-year period, and received 797 and 1,415 citations on these items), a qualitative analysis provides a more accurate picture.

Figure 1. Between 2013 and 2018, women researchers in the three schools of the Faculty of Computing and Engineering had a lower average number of publications per person (source: Scopus).

Figure 2. Between 2013 and 2018, on average women researchers in the three schools of the Faculty of Computing and Engineering received fewer citations per person (source: Scopus).

Figure 3. Between 2013 and 2018, women researchers in the three schools of the Faculty of Computing and Engineering were involved in fewer international collaborations (source: Scopus).

## **Information sheet and informed consent form**

**Research study title: Gender and research output at Dublin City University**

**REC Reference: DCUREC/2019/081**

**What is this research about?**

This research aims to explore gender disparities in academic publications of scholars at Dublin City University (DCU). The research will specifically look at the Faculty of Computing and Engineering. The principal investigator is Mr. Mohammad Hosseini.

The results of the first part of this study that consisted of a comparison of the publication record of men and women researchers at DCU showed that women tend to publish fewer peer-reviewed articles, receive fewer citations, occupy lower positions in the byline and participate less often in international projects than men. However, our understanding of the contributing factors to these differences is relatively low, and lacks local factors. Exploring factors that contribute to these disparities will have positive implications for working conditions of current employees, and recruitment of future researchers. Therefore, it is hoped that interviews of academic staff will provide a more detailed understanding of gender disparities at DCU.

**What does the research require me to do?**

I volunteer to participate in a research project conducted by Mohammad Hosseini from Dublin City University. I will be interviewed in person. However, if a meeting on campus makes me feel uncomfortable, the interview can also be arranged via online calling systems through applications such as Skype. I will be answering a series of questions related to gender disparities and my work environment.

**Please complete the following (Circle Yes or NO for each question)**

I have read the Plain Language Statement (or had it read to me)?

Yes/No

I understand the information provided?

Yes/No

I have had an opportunity to ask questions and discuss this study?

Yes/No

I have received satisfactory answers to all my questions?

Yes/No

I understand that my participation in this project is voluntary and that I will not be paid for my participation. I may withdraw from the research study at any point. If I decline or withdraw from the study the data will be immediately destructed.

Yes/No *Research and Innovation Support*

I understand that the interview is thought-provoking and could get personal. If, however, I feel uncomfortable in any way during the interview session, I have the right to decline to answer any question or to end the interview, and ask the interviewer to delete the tape.

Yes/No

I understand that notes might be written during the interview. The interview will be audiotaped and subsequently transcripts will be made for publication in a peer-reviewed journal. If I don’t want to be taped, I understand that only notes will be taken.

Yes/No

I understand that the research will not identify me by my name in any reports or publications using information obtained from the interview and that my confidentiality as a participant will remain secure. The use of records and data will be subject to standard data protection policies which protect the anonymity of participants. I understand that the confidentiality of information is subject to legal limitations.

Yes/No

I would like to receive information about the research and its results once the study is finished. I understand that I will need to provide my email that will be securely stored.

Yes/No

I have read and understood the information in this form. The interviewer has answered my questions and concerns and I have a copy of this consent form. Therefore, I consent to take part in this research project.

Yes/No

Participant Signature: ________________________________________________________

Name:

Date:

## **Questionnaire**

**Short introduction of the interviewer.**

**Introduction of the project:**

In the course of our research, we have identified some gender disparities in research output at Dublin City University. Our research has shown that women tend to publish fewer peer-reviewed articles, and are less often involved in international collaborations. For instance, we have come to understand that in the past five years, you have published … articles, and were involved in … international collaborations. As our analysis shows, you have published … times with men and … times with women authors. Most of the people you published with were from … university based in … (country). Our aim today is to ask you some further questions in order to add context to our quantitative results.

**Questions**

1. To start with, I’d like to ask whether you are satisfied with the number of papers that you publish per year?

- What will make it possible for you to publish more?
- Get better positions in the byline?
- Get more citations
- Collaborate with more international institutions

2. Our results show that on average, men publish more than women researchers.

- Do you think that this is because different demands are placed on them?
- In collaborative projects within DCU, do you feel that men and women PIs have different expectations or requirements in terms of adding co-authors to the byline?
- Do you think that you receive all the necessary information related to various projects?
- Do you think that there are smaller friendly communities with tighter bonds and friendly relationships that often collaborate and cite each other’s work?

2. Based on your experience in DCU, do you think that men and women are treated equally in the following areas?

- Enablers
  - Workload expectations
  - Academic/non-academic support
  - Access to lab/technical resources
  - Chances for travelling
- Publications
  - Opportunities for conference attendance, e.g. funding, time to travel
  - Support for journal publications, e.g. writing/editorial support, feedback from supervisors
  - Involvement in collaborative works
  - Authorship order
- Impact enhancers
  - Receiving citations
  - Opportunities for publications in Open Access journals, e.g. receive funding to pay article processing charges
  - Opportunities for publications in journals with higher impact factor, e.g. supervision and feedback
  - Collaboration in large teams
  - Collaboration in international projects
  - Collaboration in multi-disciplinary projects

3. In case you were employed elsewhere before DCU, can you provide a comparison?

4. Can you make independent and autonomous decisions about your publications or are you told/pushed to make certain decisions?

- Can you choose your co-authors to work with?
- Were there times that you were unhappy about the authors who were listed in the byline, or the order of names?

5. Follow-up questions:

- Would you encourage men/women friends or family members to start a career in your discipline?
- Would you encourage men/women friends or family members to join DCU?
- What do you think about the government initiative to create 45 women-only senior academic roles within the higher education sector over the next three years?
- Do you have any other issues/points in relation to gender disparity in your workspace that was not mentioned in this interview?

6. We are interested in your views and experiences of gender equality. After hearing below statements, please respond with one of the following options: strongly agree; agree; neither agree nor disagree; disagree; strongly disagree.

- Within my daily responsibilities, there are tasks that distract or stress me.
- Apart from teaching/supervision, all meetings, seminars, workshops and social gatherings that I am required to attend or might wish to attend, take place on weekdays between 10 am and 4 pm.
- There is a lot of pressure on me to work late or at weekends to stay on top of my work.
- I can discuss sexism with my mentor/head of school, should there be something to discuss.
- I believe DCU senior staff act as visible role models for gender equality.
- The lower position of women in higher education reduces their opportunities for publication.
- Part-time and full-time academics are given the same support for publications.
- Opportunities for publications are mainly affected by faculty and school’s politics.
- Opportunities for publications are mainly affected by the personal efforts of researchers.
- Social/educational/training activities in DCU are equally welcoming to both men and women.
- In DCU, men and women are supported equally in publishing their work.
- I have a good work/life balance.
- I feel that DCU is a great place to work for women.
- I feel that DCU is a great place to work for men.
- I have the same opportunity as my men colleagues to be involved in international collaborations.

7. We would like to ask a few demographic questions from you. (Please note that we will anonymize all our interviews and the personal information that we receive from our participants will be treated as confidential).

- Age?
- Supervisor/manager’s gender?
- Years since finished PhD, AND, since when are you employed in DCU
- Full/part-time contract?
- EU/Non-EU national?
- Marital status/Children?

## **COREQ checklist**

In this section, we report the used methodology using the checklist suggested by Consolidated criteria for reporting qualitative research (COREQ).^[20]^

1. Interviewer/facilitator: Mohammad Hosseini (M.H.) conducted all the interviews.

2. Credentials: M.H. has a PhD in Research Ethics and Integrity and Shiva Sharifzad (S.S.) has an MA in Human Rights Law.

3. Occupation: M.H. is a postdoctoral researcher in ethics and S.S. is a program assistant of gender in humanitarian action.

4. Gender: M.H. identifies as a man and S.S. identifies as a woman.

5. Experience and training: M.H. is a trained researcher with previous interview and qualitative analysis experience. S.S. is a gender equality specialist in an international organization.

6. Relationship established: M.H. has completed a PhD in Dublin City University but prior to interviews he only knew one interviewee. None of the interviewees knows S.S.

7. Participant knowledge of the interviewer: Only one interviewer knew M.H. before the interview.

8. Interviewer characteristics: M.H. introduced himself in the beginning of each interview and explained goals and objectives of the study.

9. Methodological orientation and theory: Inductive approach as suggested by Thomas, A General Inductive Approach for Analysing Qualitative Evaluation Data (2006).

10. Sampling: purposive.

11. Method of approach: The Heads of Schools of Computing, Mechanical and Manufacturing Engineering, and Electrical Engineering sent bulk invitations on M.H.’s behalf. After two weeks M.H. contacted women candidates using the available list of employees and researchers on schools’ websites. Using the public list of researchers affiliated with each school, direct email invitations were sent to thirty-three women researchers.

12. Sample size: Sixteen women were interviewed.

13. Non-participation: N/A.

14. Setting of data collection: DCU, in a building other than where candidates were based.

15. Presence of non-participants: N/A.

16. Description of sample: Women researchers (PhD, Postdoc, assistant/associate/full professor) affiliated with the Faculty of Computing and Engineering.

17. Interview guide: The questionnaire was designed by M.H. and revised according to the feedback received from the head of DCU Equality Office (Ms Sandra Healy), the Dean of the Faculty of Computing and Engineering (Professor Lisa Looney) and an external academic advisor (Dr Karen Kelsky) in 2019. Subsequently, the questionnaire was tested in a pilot (with Dr Melrona Kirrane).

18. Repeat interviews: N/A.

19. Audio/visual recording: All interviews were audio taped.

20. Field notes: N/A.

21. Duration: Interviews varied in length from 22:29 to 53:45 minutes and lasted an average of 36:13 minutes.

22. Data saturation: Saturation was not discussed but given the small number of women researchers in the faculty, the sample size was deemed reasonable.

23. Transcripts returned: No.

24. Number of data coders: Three, including both authors and a non-author contributor who had to leave the project due to personal reasons related to COVID-19 restrictions in Ireland.

25. Description of the coding tree: The first version of the codebook was developed after analysing three randomly selected interviews. From this analysis fifteen codes emerged, most of which were either renamed or merged with other codes. These included:

1. Academy versus industry: The differences in workplace environments for women.
2. Treatment of men versus women: Instances where men and women are treated differently.
3. Tendencies and preferences of men versus women: Gender specific characteristics and tendencies.
4. Bad experiences

Interviewee: Gender-based experienced that happened to the interviewee.

Others: Gender-based experienced that happened to someone the interviewee knows.

1. Gender-based roles: Tasks and roles only expected of women.
2. Exposure: Communication of work with others.
3. Family: The impact of having a family on women’s professional life and choices.
4. Funding: The impact of funding on publication record.
5. International collaborations: The impact of international collaborations on women’s publication record.
6. Nationality: The impact of nationality on women’s publication record.
7. Networking: The impact of internal and external connections on women’s publication record.
8. The need to prove themselves: Instances where women feel that they should do more.
9. Self-esteem: Instances where women’s self-esteem impacts their publication record.
10. Supervisors: Instances where working with men or women supervisors are reporter to impact women’s publication record.
11. Unwritten rules: Unofficial academic norms that affect women researchers’ publication record.

In analysing interviews, the fifteen initial codes were revised several times. Ultimately, the authors agreed on subsuming nine codes under two categories:

1. Factors that hinder women’s engagement in research publication: Issues referring to the challenges and problems that women researchers face in publishing their work.

- Gender roles: Women being assigned different tasks or assumed to have certain characteristics, thereby impacting their publication record.
- Implicit gender biases: Women being treated differently by supervisors or colleagues, affecting their publication record.
- Negative perceptions of women’s expertise and accomplishments: Instances where women’s research capabilities and academic achievements are undermined/downplayed.
- High professional standards: Instances where women's standards about their work (e.g., quality) and career choices affect their publication record.
- Family: Instances where family responsibilities such as motherhood affect women researchers’ publication record.
- Nationality: Instances where nationality affects women researchers’ career and publication record.

2. Factors that support women’s engagement in research publication: Issues that women identify as important in improving their publication record.

- Networking and research communication: Views about how networking and communication affect women’s publication record.
- Collaboration with other institutions: Views about how collaboration with other institutions affect women’s publication record.
- Funding acquisition: Views about how funding opportunities affect women’s publication record.

26. Derivation of themes: Codes were identified from the data and were not predefined.

27. Software: N/A.

28. Participant checking: N/A.

29. Quotations presented: Where relevant, direct quotations are used.

30. Data and findings consistent: We sought feedback from an external reviewer (Professor Samuel Bruton).

31. Clarity of major themes: we presented nine more commonly identified codes.

32. Clarity of minor themes: N/A

## **Results of the Likert type questionnaire**

At the end of each interview, the interviewer read the following sentences and asked the interviewee to react using five Likert type responses (i.e., strongly agree, agree to some extent, neither agree nor disagree, disagree to some extent, strongly disagree). The summary of interviewees’ responses is presented in Table 1.

Table 1. Interviewees’ responses to the Likert type questionnaire. Each cell reports the corresponding number of respondents. SA—strongly agree, A—agree to some extent, N—neutral (neither agree nor disagree), D—disagree to some extent, SD—strongly disagree.

| **Statement** | SA | A | N | D | SD | Median |
| --- | --- | --- | --- | --- | --- | --- |
| Within my daily responsibilities, there are tasks that distract or stress me. | 4 | 8 | 1 | 2 | 1 | A |
| Apart from teaching/supervision, all meetings, seminars, workshops and social gatherings that I am required to attend or might wish to attend, take place on weekdays between 10 am and 4 pm. | 4 | 5 | 0 | 4 | 3 | A |
| There is a lot of pressure on me to work late or at weekends to stay on top of my work. | 3 | 4 | 2 | 6 | 1 | N |
| I can discuss sexist practices with my mentor/head of school, should there be something to discuss. | 2 | 11 | 2 | 1 | 0 | A |
| I believe DCU senior staff act as visible role models for gender equality. | 2 | 7 | 2 | 3 | 2 | A |
| The lower position of women in higher education reduces their opportunities for publication. | 4 | 4 | 5 | 3 | 0 | A |
| Part-time and full-time academics are given the same support for publications. | 0 | 6 | 9 | 1 | 0 | N |
| Opportunities for publications are mainly affected by faculty and school’s politics. | 3 | 1 | 3 | 8 | 1 | D |
| Opportunities for publications are mainly affected by the personal efforts of researchers. | 2 | 11 | 0 | 1 | 2 | A |
| Social/educational/training activities in DCU are equally welcoming to both men and women. | 2 | 10 | 4 | 0 | 0 | A |
| In DCU, men and women are supported equally in publishing their work. | 2 | 10 | 4 | 0 | 0 | A |
| I have a good work/life balance. | 0 | 8 | 3 | 4 | 1 | A |
| I feel that DCU is a great place to work for women. | 0 | 13 | 3 | 0 | 0 | A |
| I feel that DCU is a great place to work for men. | 3 | 9 | 4 | 0 | 0 | A |
| I have the same opportunity as my men colleagues to be involved in international collaborations. | 2 | 10 | 3 | 1 | 0 | A |
